# The development of agoraphobia is associated with the symptoms and location of a patient's first panic attack

**DOI:** 10.1186/1751-0759-6-12

**Published:** 2012-04-11

**Authors:** Naomi Hara, Yukika Nishimura, Chika Yokoyama, Ken Inoue, Atsushi Nishida, Hisashi Tanii, Motohiro Okada, Hisanobu Kaiya, Yuji Okazaki

**Affiliations:** 1 Department of Psychiatry, Division of Neuroscience, Graduate School of Medicine, Mie University, Mie, Japan; 2 Department of Public Health, Fujita Health University School of Medicine, Aichi, Japan; 3 Department of Schizophrenia Research, Tokyo Institute of Psychiatry, Tokyo, Japan; 4 Research Center for Panic Disorder, Nagoya Mental Clinic, Aichi, Japan; 5 Tokyo Metropolitan Matsuzawa Hospital, Tokyo, Japan; 6 Department of Psychiatry, Division of Neuroscience, Graduate School of Medicine, Mie University, 2-174, Edobashi, Tsu, Mie 514-8507, Japan

**Keywords:** Place of first panic attack, Panic attack symptoms, Subtype of panic disorder, Agoraphobia

## Abstract

**Background:**

The place where a patient experiences his/her first panic attack (FPA) may be related to their agoraphobia later in life. However, no investigations have been done into the clinical features according to the place where the FPA was experienced. In particular, there is an absence of detailed research examining patients who experienced their FPA at home. In this study, patients were classified by the location of their FPA and the differences in their clinical features were explored (e.g., symptoms of FPA, frequency of agoraphobia, and severity of FPA).

**Methods:**

The subjects comprised 830 panic disorder patients who were classified into 5 groups based on the place of their FPA (home, school/office, driving a car, in a public transportation vehicle, outside of home), The clinical features of these patients were investigated. Additionally, for panic disorder patients with agoraphobia at their initial clinic visit, the clinical features of patients who experienced their FPA at home were compared to those who experienced their attack elsewhere.

**Results:**

In comparison of the FPAs of the 5 groups, significant differences were seen among the 7 descriptors (sex ratio, drinking status, smoking status, severity of the panic attack, depression score, ratio of agoraphobia, and degree of avoidance behavior) and 4 symptoms (sweating, chest pain, feeling dizzy, and fear of dying). The driving and public transportation group patients showed a higher incidence of co-morbid agoraphobia than did the other groups. Additionally, for panic disorder patients with co-morbid agoraphobia, the at-home group had a higher frequency of fear of dying compared to the patients in the outside-of-home group and felt more severe distress elicited by their FPA.

**Conclusion:**

The results of this study suggest that the clinical features of panic disorder patients vary according to the place of their FPA. The at-home group patients experienced "fear of dying" more frequently and felt more distress during their FPA than did the subjects in the other groups. These results indicate that patients experiencing their FPA at home should be treated with a focus on the fear and distress elicited by the attack.

## Background

In recent years, panic disorder (PD) has been recognized as a chronic disease where patients show little spontaneous improvement and disease progression is not necessarily uniform [1-7]. Agoraphobia (AG) is an anxiety symptom involving the fear of being in places or situations from which escape might be difficult (or embarrassing) or in which help may not be available in the event of an unexpected or situationally predisposed panic attack (PA) or panic-like symptoms. Agoraphobic fears typically involve characteristic clusters of situations that include being alone outside the home; being in a crowd or standing in a line; being on a bridge; and traveling in a bus, train, or automobile.

PA and the development of AG appear to have a clear linkage, a concept upheld by current biological and psychological models of PD and AG [8,9]. Early predictors for the development of AG would be important for clinical practice [10] because co-morbid AG results in poor outcomes and/or more severe PD [7,11-13]. The earliest possible predictors of the development of AG by PD patients are the features of the first panic attack (FPA) [10]. One of the key features of a patient's FPA is the location or situation in which the individual experienced their FPA.

The relationship between the situation in which the FPA occurred and the subsequent development of AG remains controversial. Some previous studies have revealed that people who experienced their FPA in public spaces [14] or phobogenic situations [15] have a high tendency to be diagnosed as having PD with AG. Shulman et al. [16] reported that extensive avoiders were more likely to have experienced their FPA in classic agoraphobic situations, such as while driving or taking public transportation. Additionally, Amering et al. [10] reported that a public occurrence of the FPA, and the accompanying feeling of embarrassment, was significantly associated with the development of AG.

On the other hand, comparisons between groups of PD patients with minimal, moderate, and marked avoidance did not demonstrate differences related to the location of the FPA or to the patients' beliefs as to what was happening to them [17]. Craske et al. [18] have reported that the various places where FPAs occurred are distributed equally between minimal and extensive avoiders. Furthermore, some of the patients who experienced their FPA at home also developed AG. According to previous studies, the percentages of PD patients, with AG, who reported FPAs at home were 8% [14], 10.3% [15], and 17% [10]. In a study comparing minimal and extensive avoiders, the percentages of extensive avoiders, who experienced their FPAs at home were 2.6% [16] and 29.4% [18], respectively.

These reports suggest an association between the location of the FPA and subsequent avoidance behaviors. However, the results remain inconclusive and investigations into other clinical factors, such as demography and symptoms experienced during the FPA, have not been conducted. For this reason, in this study, we classified the location of the FPA and explored the differences in clinical features (e.g., symptoms of the FPA, frequency of AG, and severity of the FPA) of five groups. Additionally, the clinical features of patients who developed AG after experiencing their FPA at home were examined. There was an absence of detailed research examining patients who experienced their FPA at home; therefore, for PD patients with AG, the clinical features of these patients were also compared to those of the patients who experienced their FPA elsewhere.

## Methods

### Subjects

The study subjects consisted of 1,075 outpatients with PD, with or without AG, who initially visited the Nagoya Mental Clinic in Nagoya, Japan, between April 1998 and September 2001 and who were diagnosed according to the criteria in the Diagnostic and Statistical Manual of Mental Disorders, 4th ed. (DSM-IV) [19]. Exclusion criteria included somatic illnesses; a co-morbidity of psychiatric disorders, except major depressive disorder; and current substance-related disorders. Of the total population, 53 patients were excluded from analysis due to a co-morbid mental disease. Thus, we analyzed the data of 1,022 patients.

This study was approved by the institutional ethical committees of the Mie University School of Medicine and the Warakukai Nagoya Mental Clinic. All subjects who participated in this study provided written, informed consent for study participation.

### Initial visit interview and assessment

FPA symptoms and location of occurrence were documented by means of a questionnaire administered during each patient's initial clinic visit. The questionnaire explained the definition of PA and described the 13 symptoms of PAs. The patients chose the symptoms that they experienced during their FPA and filled in the date and location of the FPA. In addition, patients answered questions regarding their age at the time of their FPA, the number of hospitals they had visited, and the length of time from the FPA to the time of consultation. The onset of PD was defined as the day on which the patient experienced his or her FPA. The patients also scored the severity of their PD (based on the severity of their FPA, where 0 = none, 1 = mild, 2 = moderate, 3 = severe, 4 = very severe); their frequency of anticipatory anxiety (0 = never, 1 = seldom, 2 = sometimes, 3 = often, 4 = always); and their degree of avoidance behaviors (0 = never, 1 = seldom, 2 = sometimes, 3 = often, 4 = always).

The Zung Self-Rating Depression Scale (SDS) is a short, 20 items, self-administered survey that quantifies the depression status of a patient [20,21]. The scale yields an overall score of 20-80, with higher scores reflecting more severe symptoms of depression.

Other demographic information was also obtained during the initial assessment, including age, gender, years of education, drinking status, smoking status, family history, and duration of illness.

### Statistical analyses

First, the locations of the FPAs were classified. Some patients (n = 192) gave incomplete responses regarding the place of their FPA; therefore, the responses of 830 people were included in the comparison analyses. The prevalence of AG in each group was investigated as was the relationship between the clinical symptoms experienced and the location of the FPA.

The relationship between the clinical features of the symptoms experienced and the place of the FPA was investigated by use of nonparametric 2 × 5 Chi-square tests of significance for categorical data (gender, PA symptoms, co-morbidity of AG, drinking status, smoking status, family history). An analysis of variance (ANOVA) was used to compare patient age, years of education, age at FPA, severity of FPA, degree of anticipation, degree of avoidance behavior, number of hospital consultations, duration of consultations, SDS score, and number of DSM symptoms. All statistical inferences were made at the 5% level of p values as significant.

In PD patients with AG, clinical features were compared between the subjects who experienced their FPA at home (n = 115) and those who experienced their FPA outside of home (n = 267). Statistical differences between the 2 groups (at home and outside of home) were assessed using Chi-square tests for categorical variables, and they were also compared relative to age, years of education, age at FPA, severity of FPA, degree of anticipation, degree of avoidance behavior, number of hospital consultations, duration of consultations, SDS score, and number of DSM symptoms by using independent sample t-tests (two-tailed). The Statistical Package for Social Sciences (SPSS 11.5) was used for all statistical analyses.

## Results

### FPA location frequency and demographics

Table 1 shows the frequency of FPAs at various places. The subjects (n = 830) were divided into 5 groups based on the place where they experienced their FPA: at home; at school/office; while driving a car; as a passenger in a public transit vehicle (automobile, train, bus, airplane); and outside of home (store, hospital, beauty salon, movie theater, on the street). The most prevalent locations where subjects experienced their FPA were at home (37%), followed by outside of home (20%) and at school/office (15%).

**Table 1:** Demographics and panic characteristics (N = 830)

Place of first PA	Total (N = 830) 100%	1 At home (N = 308) 37.10%	2 office, school (N = 128) 15.40%	3 driving (N = 100) 12.00%	4 train, bus (N = 128) 15.40%	5 out of home (N = 166) 20.00%	statistical value (F or chi-square)	p-value
Age	34.60 ± 10.01	35.42 ± 10.76	33.77 ± 10.76	33.70 ± 8.18	34.27 ± 9.48	34.54 ± 9.32	0.974	0.421

Sex (M/F)	300 · 530(36.1% · 63.9%)	88 · 220(28.6% · 71.4%)	61 · 67(47.7% · 52.3%)	49 · 51(49.0% · 51.0%)	45 · 83(35.2% · 64.8%)	57 · 109(34.3% · 65.7%)	22.452	< 0.001

Years of Education	13.13 ± 2.20	13.06 ± 2.15	13.06 ± 2.49	13.32 ± 2.10	13.45 ± 2.07	12.95 ± 2.20	1.245	0.29

Drinking (No/Yes)	508 · 288(63.8% · 36.2%)	203 · 88(69.8% · 30.2%)	72 · 54(57.1% · 42.9%)	52 · 43(54.7% · 45.3%)	70 · 55(56.0% · 44.0%)	111 · 48(69.8% · 30.2%)	16.06	0.003

Smoking (No/Yes)	457 · 283(61.8% · 38.2%)	176 · 91(65.9% · 34.1%)	73 · 42(63.5% · 36.5%)	38 · 48(44.2% · 55.8%)	70 · 48(59.3% · 40.7%)	100 · 54(64.9% · 35.1%)	14.298	0.006

Family history	531 · 284(65.2% · 34.8%)	192 · 112(63.2% · 36.8%)	85 · 43(66.4% · 33.6%)	66 · 30(68.8% · 31.3%)	86 · 42(67.2% · 32.8%)	102 · 57(64.2% · 35.8%)	1.472	0.823

**First Panic Attack**								

Age at first PA	28.96 ± 9.75	29.99 ± 10.25	27.77 ± 11.28	29.29 ± 7.87	27.84 ± 7.84	28.66 ± 9.79	1.823	0.122

Severity (0-4)	3.12 ± 0.92	3.22 ± 0.80	2.84 ± 1.03	3.20 ± 0.88	3.01 ± 1.00	3.17 ± 0.94	3.757	0.005

**Consultation behavior**								

Consultation hospitals (n)	2.51 ± 2.08	2.59 ± 1.81	2.46 ± 1.81	2.40 ± 3.24	2.16 ± 1.60	2.72 ± 2.18	1.517	0.195

Duration of consultation to psychiatric service (days)	2105.48 ± 2558.39	2015.35 ± 2573.48	2300.57 ± 2746.20	1648.10 ± 1812.51	2377.37 ± 2600.39	2187.63 ± 2710.60	1.488	0.204

**At the initial visit**								

Agoraphobia (% of each group)	382(46.0%)	115(37.3%)	52(40.6%)	56(56.0%)	78(60.9%)	81(48.8%)	26.836	< 0.001

Depression (SDS score)	40.69 ± 12.04	40.65 ± 11.66	42.71 ± 12.21	38.66 ± 12.49	38.59 ± 12.33	42.11 ± 11.82	2.596	0.035

Anticipation anxiety (0-4)	3.08 ± 1.10	3.00 ± 1.15	3.01 ± 1.08	3.00 ± 1.10	3.14 ± 1.06	3.28 ± 1.00	.651	0.16

Avoidance (0-4)	2.30 ± 1.45	2.04 ± 1.46	2.34 ± 1.46	2.54 ± 1.41	2.46 ± 1.50	2.48 ± 1.38	4.077	0.003

Table 1 also presents the socio-demographic data of the subjects in the 5 groups. There were significant differences in the gender ratio (*p *< 0.001), drinking status (*p *= 0.002), and smoking status (*p *= 0.006), among the 5 groups of patients at their initial clinic visit.

### FPA symptom profiles among the 5 groups

Table 2 shows the FPA symptom profiles among the patients in the 5 groups. Of the 13 FPA symptoms, sweating (p = 0.005), chest pain (*p *= 0.010), feeling dizzy (*p *= 0.017), and fear of dying (*p *< 0.001) were those most frequently experienced by the individuals in the public transit group, school/office group, driving group, and at-home group, respectively. Additionally, the at-home group experienced more severe distress caused by their FPA, while the office group experienced the least severe distress.

**Table 2:** Frequency of Symptoms at first panic attack (N = 830)

Place of first PA		Total	1 At home	2 office, school	3 driving	4 train, bus	5. outside of home	statistical value	p-value (place of 5 group)
			
		(N = 830)	(N = 308)	(N = 128)	(N = 100)	(N = 128)	(N = 166)	F or chi-square	
First PA symptom (DSM-IV), N(%)									

I	Palpitations	745 (89.8)	273 (88.6)	117 (91.4)	89 (89.0)	116 (90.6)	150 (90.4)	1.033	0.905

II	Sweating	420 (50.7)	134 (43.6)	69 (53.9)	59 (59.0)	78 (60.9)	80 (48.5)	15.081	0.005

III	Trembling	426 (51.3)	167 (54.2)	63 (49.2)	58 (58.0)	56 (43.8)	82 (49.4)	6.231	0.183

IV	Smothering	597 (71.9)	229 (74.4)	88 (68.8)	72 (72.0)	85 (66.4)	123 (74.1)	3.855	0.426

V	Feeling of choking	402 (48.4)	157 (51.0)	53 (41.4)	49 (49.0)	60 (46.9)	83 (50.0)	3.627	0.459

VI	Chest pain	237 (28.6)	98 (31.8)	45 (35.2)	21 (21.0)	24 (18.8)	49 (29.5)	13.247	0.01

VII	Nausea	273 (32.9)	95 (30.8)	43 (33.6)	37 (37.0)	51 (39.8)	47 (28.3)	5.757	0.218

VIII	Feeling dizzy	431 (51.9)	143 (46.4)	71 (55.5)	60 (60.0)	59 (46.1)	98 (59.0)	12.09	0.017

IX	Derealization	250 (30.1)	92 (29.9)	43 (33.6)	33 (33.0)	35 (27.3)	47 (28.3)	1.863	0.761

X	Going crazy	428 (51.7)	170 (55.4)	55 (43.0)	59 (59.0)	65 (50.8)	79 (47.9)	8.71	0.069

XI	Fear of dying	454 (54.7)	195 (63.3)	51 (39.8)	56 (56.0)	62 (48.4)	90 (54.2)	22.729	< 0.001

XII	Paresthesia	248 (29.9)	105 (34.1)	34 (26.6)	29 (29.0)	36 (28.1)	44 (26.5)	4.406	0.354

XIII	Chills or hot flushes	247 (29.8)	101 (32.8)	33 (25.8)	23 (23.0)	37 (28.9)	53 (31.9)	4.928	0.295

Total number (1-13)		6.21 ± 2.82	6.33 ± 2.78	5.99 ± 2.87	6.50 ± 2.67	5.97 ± 3.05	6.19 ± 2.77	0.826	0.509

### FPA location and ratio of co-morbidity with AG at each FPA location

The groups of individuals who experienced their FPA while in a public transit vehicle (60.9%) or while driving (56.0%) had significantly higher ratios of co-morbid AG than did other groups (Table 1). In each diagnostic group, the PD patients with AG tended to experience their FPA while driving or in a public transit vehicle, while the PD patients without AG tend to experience their FPA at home (Table 1). With respect to subjective depressive symptoms, as assessed by the SDS, individuals in the school/office group and the outside-of-home group showed the highest scores, whereas the driving group and the public transit group showed the lowest scores (*p *= 0.033, Table 1). The degree of avoidance behavior reported by patients at their initial visit was least for the patients in the at-home group (*p *= 0.003, Table 1).

### Clinical characteristic differences between the at-home and outside-the-home patients with AG

Tables 3 and 4 show the socio-demographic data and symptom profiles of the FPAs of the PD patients included in the at home and outside-of-home groups who also had co-morbid AG. The at-home group, compared to the outside-of-home group, had a higher proportion of women (*p *= 0.022) and experienced more severe distress elicited by their FPA (*p *= 0.010). Furthermore, of the 13 FPA symptoms, the at-home group subjects reported a higher frequency of fear of dying compared to those in the outside-of-home group (OR = 0.37 (0.23-0.59), *p *< 0.001).

**Table 3:** Demographics and panic characteristics (N = 382, mean ± SD)

Place of first PA	at home	outside of home	statistical value	p-value	OR	95% Confidence Interval	
						
	(N = 115)	(N = 267)	F or chi-square			Lower	Upper
Age	35.37 ± 10.03	33.95 ± 8.79	3.86	0.189			

Sex (M/F)	26/89 (22.6%/77.4%)	92/175 (34.5%/65.5%)	5.29	0.022	0.556	0.335	0.920

Years of Educatiion	13.0 ± 2.16	13.21 ± 2.29	1.24	0.431			

Drinking (No/Yes)	72/34 (67.9%/32.1%)	164/94 (63.6%/36.4%)	0.63	0.470	1.214	0.751	1.962

Smoking (No/Yes)	67/38 (63.8%//36.2%)	149/98 (60.3%/39.7%)	0.38	0.552	1.160	0.723	1.860

Family history (No/Yes)	78/36 (68.4%/31.6%)	177/82 (68.3%/31.7%)	0.00	1.000	1.004	0.625	1.612

**First Panic Attack**							

Age at first PA	28.39 ± 9.14	27.40 ± 7.96	2.05	0.286			

Severity (0-4)	3.31 ± 0.74	3.06 ± 0.98	6.03	0.010			

Total number (1-13) of FPA symptons	6.64 ± 2.53	6.12 ± 2.80	3.56	0.083			

**Consultation behavior**							

Consultation hospitals (n)	2.91 ± 2.11	2.54 ± 2.12	0.33	0.116			

Duration of consultation to psychiatric service (days)	2552.15 ± 2748.88	2474.13 ± 2641.34	1.05	0.794			

**At the initial visit**							

Depression (SDS score)	41.24 ± 10.98	40.63 ± 11.89	0.22	0.669			

Anticipation anxiety (0-4)	3.23 ± 1.08	3.32 ± 0.92	3.38	0.445			

Avoidance (0-4)	2.90 ± 1.08	2.83 ± 1.30	8.80	0.592			

**Table 4:** At home group vs outside of home group in PD with AG (N = 382)

Item		Odds Ratio (95%CI)	_2	p-value	Experience rate	
					
					At home (N = 115)	Outside of home
I	Palpitations	1.78 (0.79-4.00)	1.974	0.19	0.90	0.94

II	Sweating	1.11 (0.72-1.72)	0.218	0.66	0.53	0.56

III	Trembling	0.87 (0.56-1.36)	0.361	0.58	0.54	0.51

IV	Smothering	0.68 (0.40-1.15)	2.088	0.16	0.80	0.73

V	Feeling of choking	0.88 (0.57-1.37)	0.311	0.66	0.52	0.49

VI	Chest pain	0.69 (0.42-1.14)	2.129	0.15	0.28	0.21

VII	Nausea	0.95 (0.60-1.51)	0.041	0.91	0.35	0.34

VIII	Feeling dizzy	1.35(0.87-2.09)	1.796	0.18	0.46	0.54

IX	Derealization	1.04 (0.65-1.67)	0.03	0.91	0.31	0.32

X	Going crazy	0.67 (0.43-1.05)	3.068	0.09	0.61	0.51

XI	Fear of dying	0.37(0.23-0.59)	18.046	< 0.001	0.70	0.47

XII	Paresthesia	0.65 (0.40-1.06)	3.035	0.10	0.31	0.23

XIII	Chills or hot flushes	0.82(0.51-1.32)	0.647	0.46	0.32	0.28

df = 1						

### Examination of gender difference

Male-to-female ratios differed in the profiles of the 5 groups. Therefore, gender differences, not location of the FPA, could possibly explain our results. To evaluate this possibility, an analysis of the 5 groups was conducted to assess gender differences with regard to the symptoms of the FPA, the severity of the FPA, the ratio of co-morbid AG, SDS scores, avoidance scores, and 4 of the FPA symptoms (sweating, chest pain, feeling dizzy, and fear of dying).

Three factors showed significant differences in the male-to-female ratio: co-morbidity of AG (M: 39.3% < F: 49.8%, p = 0.004), chest pain (M: 33% > F: 26%, p = 0.038), and fear of dying (M: 59.3% > F: 52.1%, p = 0.050). For these 3 factors, we performed the comparison among 5 groups based on the places each gender experienced FPA. With regard to the co-morbidity of AG, a significant difference was observed among males (p = 0.003) and females (p = 0.001); for chest pain, a significant difference was observed only among males (p = 0.027); for fear of dying, there was a significant difference only among females (p < 0.001) who experienced this symptom within the at-home group. However, the tendency of each gender was almost the same as the total sample, as shown in Table 1.

## Discussion

The purpose of this study was to explore the clinical features of FPAs, based on their location of occurrence. This was accomplished by making comparisons among 5 groups of patients, classified by the place of their FPAs. These comparisons revealed differences among 7 descriptors (sex ratio, drinking status, smoking status, FPA symptom severity, SDS score, frequency of co-morbid AG, and degree of avoidance behavior) and 4 symptoms (sweating, chest pain, feeling dizzy, and fear of dying) associated with the FPA.

People who experienced their FPA in a public transit vehicle or while driving demonstrated a higher frequency of co-morbid AG and the highest degree of avoidance behavior; these results are in keeping with those of previous studies [10,14-16,18]. The present study also confirmed that the place of the FPA correlated to the development of AG. Of the patients diagnosed with PD and co-morbid AG, those who experienced their FPA at home more frequently experienced a fear of dying during their FPA and felt more distress than those in the group that experienced their FPA outside the home. Thus, the patients in these 2 groups are considered to have different clinical features.

### At-home group

A smaller proportion of people who experience their FPA at home subsequently developed AG. However, a significant proportion of individuals within this group (37%) still developed AG. The results show a similar frequency as has been reported in previous studies [10,14-16,18].

In this study, patients in the at-home group experienced a fear of dying more frequently than did patients in any other group. This finding suggests that fear of dying might be a characteristic symptom of people who experience their FPA at home. Vickers and McNally (2005) [22] reported that the fear of dying is the symptom that best distinguishes the panic attacks of individuals diagnosed with PD from those without PD. In their study, the fear of dying had the largest effect size, an association with PD that persisted after control for other symptoms, and a continued importance after multivariate analyses. Additionally, Segui et al. [23] reported that palpitations (86.7%), shortness of breath (76.5%), fear of dying (69.9%), and dizziness (63.6%) were the most frequent and intense symptoms reported by PD patients. Cox et al. [24] also reported that, of the DSM symptoms, fear of dying and tachycardia were the symptoms most often rated as very severe. The findings in the current study suggest that patients experiencing the most severe symptoms during their FPA were those who experienced that attack at home. The fact that the at-home group of patients experienced more severe symptoms during their FPA than did those in other groups, suggests that early interventional treatment of PD may be particularly important to the patients in the at-home group.

### FPA symptoms and the location of the FPA

Other than fear of dying, significant differences were found for 3 other symptoms and were related to the location of the FPA. A higher proportion of patients in the driving and public transit groups experienced sweating. In clinical practice, many PA patients describe this symptom, including descriptions of experiences such as wet hands while driving. Some studies report that such autonomic occurrences may be a symptom subtype that is often mixed into other groups of physical symptoms.

Chest pain was a physical symptom experienced by a higher proportion of the patients who had their FPA while at school/office as well as by those in the at-home group. Lang et al. [25] reported that chest pain or discomfort occurred more often in cases of PD without AG. The school/office group and the at-home group had the lowest rate of AG, suggesting that the current results are consistent with those of the Lang et al. [25] report.

Feeling dizzy was reported by a higher proportion of the patients classified into the driving and outside home groups. Several studies have reported a relationship between feeling dizzy and AG. Yardley et al. [26] studied the prevalence of PD symptoms in a sample of patients experiencing dizziness and examined how this affects them psychosocially. Patients with panic-related dizziness were reported to have higher rates of vertigo and agoraphobic behavior when compared to those patients who had only panic or dizziness alone. Jacob et al. [27] reported that vestibular symptoms during panic spells are not necessarily related to the presence of vestibular dysfunctions that are objectively identifiable; nonetheless, patients with PD associated with AG have significantly more vestibular disorders than other patients. Vaillancourt and Bélanger [28] also reported that people suffering from both PD and dysfunctions of the equilibrium system might avoid activities that rely heavily upon good balance; such as walking on uneven surfaces or undertaking some forms of transportation. Thus, the driving group, as well as the outside-of-home group, which demonstrated higher rates of AG had been active outside of the home (e.g., walking on the street) in an adverse situation and experienced a higher frequency of feeling dizzy.

### Other factors

Other demographic characteristics also show significant differences when classified according to the reported place where the patient's FPA was experienced. The at-home group showed a high proportion of females, while the school/office and driving groups showed a high proportion of males. As might be expected, the place where a particular group of people spends the majority of their daily life influences this result (for example, there are many women who remain at home and a higher proportion of men who spend significant amounts of time driving and at work).

A higher proportion of people in the school/office, driving, and public transit groups reported alcohol consumption than in other groups. Interestingly, PD occurs at a higher rate among alcohol abusers [29,30]. Craske et al. [18] found that the majority of their PD subjects reported only 1 life stressor and it was often related to ill-health or alcohol use.

A far higher proportion of subjects in the driving group were reported to be smokers. This group was also observed to have a higher proportion of men; therefore, there may be a relationship between male smokers and PD. Pohl et al. [31] reported that the prevalence of smoking was significantly higher in female PD patients than for control subjects (40% vs. 25%). However, smoking rates among males did not differ between PD patients and control subjects. Other studies have reported that patients with anxiety disorders have a greater propensity to be smokers [32,33]. These inconsistencies suggest that further study regarding the association between smoking and AG are required.

## Limitations

There were several limitations to this study. First, it was difficult to define the timing of some individuals' FPA. People who had experienced their FPA during childhood had difficulty remembering the experience in detail. As a result, there were a large number of blanks when describing the place, situation, or symptoms of the FPA. This led to nearly 200 responses being excluded from the final data analysis. In contrast, because the FPA was a high-impact event, many occurrences were described in detail and are believed to be reliable. The finding that a great majority of PD patients are able to vividly describe their FPA was first described by Lelliot [14] and confirmed in a later study [10].

Second, like all previous studies describing the onset of PDs, the present one was necessarily retrospective. The main problem in studying the onset of PD is the difficulty associated with prospectively studying large cohorts of subjects, as prodromal symptoms of anxiety disorders are highly prevalent in the general population [34]. With this limitation in mind, the best strategy is to recruit patients at the first stage of illness, after the initial consultation with emergency services or general practitioners. This approach would allow for the opportunity to analyze patient clinical features before the start of treatment and to follow them prospectively.

Third, a gender difference was observed with regard to the place of the patient's FPA, suggesting that an individual's daily routine influences the location of their FPA. In this study, men and women demonstrated similar tendencies toward symptoms within each location of the FPA. However, further investigation into the impact of gender differences and lifestyles on FPA symptoms are required.

## Conclusion

The purpose of this study was to determine the association between the clinical features and location at which an individual experiences his or her FPA and the development of AG. The present study shows that the public transit vehicle and driving groups have a high tendency to demonstrate co-morbid AG. The result suggests that the PD patients who experienced FPA in a public transit vehicle or while driving might be monitored with particular attention to co-morbidity of AG at every visit. Additionally, the at-home group experienced distinctly different clinical features as compared to those whose FPA occurred in outside-of-home locations. The at-home group of patients experienced "fear of dying" symptoms more frequently and felt more distress during their FPA. The results indicate that patients experiencing their FPA at home should be treated with a focus on the fear and distress elicited by their FPA. We conclude that PD is heterogeneous, and the further examinations are needed in order to provide a specific intervention to meet individual symptoms.

## Abbreviations

PD: panic disorder; FPA: first panic attack; AG: agoraphobia; DSM-IV: the Diagnostic and Statistical Manual of Mental Disorders: 4th ed.; SDS: The Zung Self-Rating Depression Scale.

## Competing interests

The authors declare that they have no competing interests.

## Authors' contributions

NH and YO coordinated the entire research design and took responsibility for the management of this study. NH, CY, KI, and HK collected the data. NH and YN analyzed and interpreted the data and AN, HT, and MO provided advice on data analysis. NH and YN drafted the manuscript. All authors contributed to the critical revision and final approval of the manuscript.
